# A genome scan for parent-of-origin linkage effects in alcoholism

**DOI:** 10.1186/1471-2156-6-S1-S160

**Published:** 2005-12-30

**Authors:** Xiao-Qing Liu, Celia MT Greenwood, Ke-Sheng Wang, Andrew D Paterson

**Affiliations:** 1 Program in Genetics and Genomic Biology, The Hospital for Sick Children, University of Toronto, Toronto, Ontario, Canada; 2 Department of Public Health Sciences, University of Toronto, Toronto, Ontario, Canada

## Abstract

**Background:**

Alcoholism is a complex disease in which genomic imprinting may play an important role in its susceptibility.

**Objective:**

To conduct a genome-wide search for loci that may have strong parent-of-origin linkage effects in alcoholism; to compare the linkage results between the microsatellites and the two single-nucleotide polymorphism (SNP) platforms.

**Methods:**

Nonparametric linkage analyses were performed using ALLEGRO with the three sets of markers provided by the Genetic Analysis Workshop 14 for the Collaborative Study on the Genetics of Alcoholism Problem 1 data. Both sex-averaged and sex-specific genetic maps were used. We also provided a valid statistical test to determine whether the parental allele sharing differed significantly.

**Results:**

Significant maternal linkage effects (paternal imprinting) were observed on chromosome 12 using either the microsatellite markers or the two SNP panels. The two SNP sets did not improve the linkage signals compared to the results from the microsatellite markers on chromosome 12. Possible paternal linkage effects (maternal imprinting) on chromosome 7 and maternal linkage effects (paternal imprinting) on chromosome 10 were found using the two SNP panels.

**Conclusion:**

For diseases which may have parent-of-origin effects, linkage analysis looking at parental sharing separately may reduce locus heterogeneity and increase the ability to identify that which can not be identified with usual linkage analysis.

## Background

Genomic imprinting (a class of parent-of-origin effects) occurs when the expression of a gene is dependent on the parent from which it was inherited. It has been suggested that genomic imprinting plays a role in the susceptibility to alcoholism (alcohol dependence) [1]. In the Genetic Analysis Workshop 11 (GAW11), using the Collaborative Study on the Genetics of Alcoholism (COGA) data, three groups incorporated parent-of-origin linkage analyses [2-4] while a fourth group performed the transmission disequilibrium test (TDT) separately for paternal and maternal transmissions [5]. Possibly due to the different approaches used, the results from the above studies did not replicate each other.

In this study, we performed nonparametric linkage analyses using the microsatellite markers and single-nucleotide polymorphisms (SNPs) from Illumina and Affymetrix to search for loci with parent-of-origin effects in alcoholism. We also proposed a new test to determine whether the paternal and maternal allele sharing were significantly different from each other.

## Methods

### Data description

The data from COGA provided for the GAW14 were used. The original dataset contains 143 extended pedigrees. We focused our study on the 112 Caucasian families. Due to computational limitations, 52 unaffected individuals who were least genotyped were removed from the 8 largest pedigrees. We used the COGA definition of alcoholism (ALDX1) as the phenotype. The affection status was coded as affected if ALDX1 was 5 (affected). All the other individuals were coded as unaffected.

All three sets of markers (328 microsatellite markers, 4,720 clean SNPs from Illumina, and 11,120 clean SNPs from Affymetrix) were used for two-point linkage analyses. Multipoint analyses were applied to all the autosomes for the microsatellite markers, 7 chromosomes (2, 7, 9, 10, 12, 13, and 16) for the Illumina SNPs, and segments of 10 chromosomes (1, 2, 4, 7, 9, 10, 11, 12, 13, and 16) for the Affymetrix SNPs based on the two-point linkage signals. Instead of using the provided marker allele frequencies, we generated them with the computer program PEDMANAGER v0.9 (MP Reeve-Daly, Whitehead Institute).

The sex-averaged and sex-specific genetic maps for the microsatellite markers were obtained from the Rutgers map [6] based on NCBI build 34. When a marker was not found in this map, the closest marker was identified by its physical location (from the UCSC Genome Bioinformatics database) and that marker's genetic location was used in the analysis. The sex-averaged and sex-specific genetic maps for the SNPs from Illumina were provided by GAW14 (NCBI build 33). The sex-averaged map for the SNPs from Affymetrix was provided by GAW14, and its sex-specific map was derived from the provided deCode genetic map (August 2001 freeze) [7]. Our results were reported in the marker panel-specific genetic locations. Caution should be taken when comparing the results from different marker panels because the maps were from different sources. For the analyses using the combination of all three marker panels on chromosome 12q, the markers were ordered according to NCBI build 34 and their genetic locations were derived from the Rutgers map [6]. Because the multipoint analysis in ALLEGRO was based on a no-interference model, the Kosambi map was converted into the Haldane map for the analyses while all results were reported in the Kosambi scale.

### Statistical methods

ALLEGRO v1.2c [8] was applied for both the two-point and multipoint linkage analyses with the exponential allele-sharing model [9]. The S_pair _(allele sharing by affected pairs) scoring function was applied in all cases. The evidence for paternal and maternal imprinting was investigated with an imprinting-based score function [10] that considers separately the paternal and maternal allele sharing of two affected relatives.

To test the null hypothesis that the maternal and paternal allele sharing are the same, we conducted a likelihood ratio test:

(*Zlr*_*m*_^2 ^+ *Zlr*_*p*_^2^) - *Zlr*_*b*_^2 ^~ *χ*_(1)_^2^,

where



*sign(dhat) *is the sign of *dhat*, the measurement of the size of genetic effect [9], and *LOD *is the allele sharing LOD score. *Zlr*_*m*_, *Zlr*_*p*_, and *Zlr*_*b *_are the *Zlr *values for maternal sharing, paternal sharing, and both parents' allele sharing, respectively. This likelihood ratio test has an asymptotic *χ*^2 ^distribution with one degree of freedom under the null hypothesis assuming that the maternal identity by descent (IBD) is independent of the paternal IBD or in the absence of linkage. This assumption of the independence of maternal and paternal allele sharing is valid under an additive genetic model.

## Results and Discussion

Table 1 shows the results from the two-point linkage analysis with all the three sets of markers. The markers that have significant parental effects (*p *≤ 0.05) with one parental LOD score ≥ 2.5 are listed. Of these markers, one microsatellite marker on chromosome 12, two SNPs on chromosomes 2 and 13 from Illumina, and one SNP on chromosome 2 from Affymetrix showed significant excess maternal allele sharing, while four SNPs on chromosomes 1, 4, 7, and 11 from Affymetrix showed significant excess paternal allele sharing.

**Figure 1:**
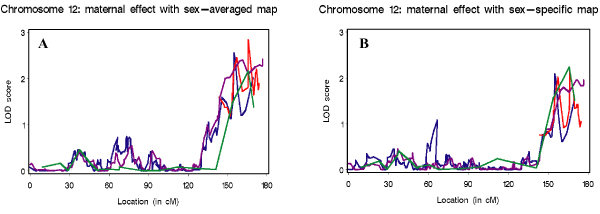
**Multipoint linkage results for maternal effect on chromosome 12. **(A) Results for maternal allele sharing using sex-averaged genetic map; (B) results for maternal allele sharing using sex-specific map. Colors: microsatellite markers (green), SNPs from Illumina (purple), SNPs from Affymetrix (blue), combination of 3 microsatellite markers, 19 SNPs from Illumina, and 28 SNPs from Affymetrix (red).

In the multipoint linkage analyses using the sex-averaged genetic maps, the same region that showed significant maternal effects on chromosome 12 using the microsatellite markers (at 165.9 cM) in the two-point analysis also showed significant maternal effects using the SNPs from both Illumina and Affymetrix (at 161.6 cM and 154.4 cM, respectively) (Table 2 and Figure 1A). Excess maternal sharing on chromosome 12 was also detected using the microsatellite markers (LOD = 2.17 with *p *= 0.0007). However, it was not significantly different from the paternal effects (parental effect *p *= 0.13). Five more loci on chromosomes 2, 7, 10, and 13 showed signals of paternal effects but none of their LOD scores was higher than 2.

In the multipoint linkage analyses using the sex-specific genetic maps, a LOD score of 2.16 (*p *= 0.0007) was observed at the same region (154.4 cM) on chromosome 12 using the SNPs from Affymetrix (Table 3). The peaks did not change dramatically when either of the two SNP panels were used compared to when the microsatellite markers were used (Figure 1B). These results are consistent with our observation that the information content at this region on chromosome 12 was similar for all three sets of markers. However, when testing for the parent-of-origin effects, the information content was lower when the sex-specific maps were used than when the sex-averaged maps were used (*p *< 0.0001). This observation was also true for all the other tested chromosomes. Because information content depends on the genetic maps through multipoint allele sharing estimation, a possible explanation for the observation is that ALLEGRO incorporates the sex-averaged maps differently from the sex-specific maps in parental effect linkage analysis.

In addition to the consistent results across the three panels of markers on chromosome 12, one region on chromosome 7 (at 1.2 cM for Illumina and at 13.7 cM for Affymetrix) and one region on chromosome 10 (at 57.0 cM for Illumina and at 62.5 cM for Affymetrix) also showed consistent signals of paternal and maternal effects, respectively, using both SNP panels (Table 3). The most telomeric microsatellite marker on chromosome 7 was located at 19.2 cM, and did not show excess paternal sharing (parental effect *p *= 0.1). For the region on chromosome 10, there was a maternal effect peak at 57.7 cM (LOD = 1.46 with *p *= 0.005) from the microsatellite markers. However, it was not significantly different from the paternal effects (parental effect *p *= 0.06).

Because all the three sets of markers showed linkage signals of excess maternal allele sharing for alcoholism on chromosome 12, we selected 3 microsatellite markers, 19 SNPs from Illumina, and 28 SNPs from Affymetrix around the most significant region. These 50 markers covered about 30 cM of the chromosome. We repeated the linkage analyses using this combined dataset. The results did not improve very much compared to the results from the individual marker set (Figure 1A, and 1B). The highest maternal effect LOD scores were 2.83 (*p *= 0.0001) using the sex-averaged map and 2.14 (*p *= 0.0008) using the sex-specific map at 165.9 cM (closest marker D12S1045). The paternal results were close to zero at this location. The parental effect *p*-values were 0.002 and 0.02 for analyses using the sex-averaged and sex-specific maps, respectively. The female/male genetic map ratio around this 30-cM region was 1.7 (ranges from 1.56 to 1.85), which did not differ from the genome-wide mean of 1.65 [7].

Given the large number of tests we conducted, some of the significant parental effects could happen by chance. In addition, the test for parental effect assumes independence of paternal and maternal allele sharing, which is true under an additive genetic model. If the genetic model is not additive, the maternal and paternal sharing will be dependent and the parental test statistic will have less than one degree of freedom. In this case, our test will be conservative and will have low power. The test for parental effect can also be affected by marker informativity. An examination of the paternal and maternal *dhat *values (the size of genetic effect) can help in this situation.

Comparing our study with the previous studies from GAW11 [2-5], our results did not replicate any of the significant results from GAW11. This is possibly due to differences in the families, genotype data, statistical methods, and genetic maps.

## Conclusion

In this study, we conducted a genome-wide scan of loci that might have significant parent-of-origin linkage effects in alcoholism. Evidence of excess maternal sharing (paternal imprinting) on chromosome 12 was shown in analyses using the microsatellite markers and the two SNP panels. In addition, the results from the two SNP panels showed evidence of excess paternal sharing on chromosome 7 and excess maternal sharing on chromosome 10. The significance was similar using the microsatellite markers versus the SNP sets due to their similar information content on chromosome 12. We also observed a drop of information content when the sex-specific maps were used in imprinting linkage analysis compared to when the sex-averaged maps were used, which might explain the lower LOD scores from our analysis using the sex-specific maps.

## Abbreviations

COGA: Collaborative Study on the Genetics of Alcoholism

GAW: Genetic Analysis Workshop

IBD: Identity by descent

SNP: Single-nucleotide polymorphism

TDT: Transmission disequilibrium test

## Authors' contributions

X-QL designed the study, performed the statistical analysis, and drafted the manuscript. CMTG provided the novel statistical method used in this study and assisted with the interpretation. K-SW helped with the revision of the draft. ADP conceived of the study and helped to draft the manuscript. All authors read and approved the final manuscript.

## Figures and Tables

**Table 1:** Linkage results of two-point analysis^a^

				LOD score (*p*-value)			
					
Marker data	Chr	Location (cM)	Marker name	Both	Maternal	Paternal	Parental effect *p*-value
Microsatellite	12	165.9	D12S1045	1.58 (0.003)	**3.17 (0.00006)**	0.05 (0.7)	0.006
Illumina	2	0	rs876724	1.06 (0.01)	**2.73 (0.0002)**	0.009 (0.6)	0.005
	13	92.8	rs141505	2.54 (0.0003)	**3.08 (0.00008)**	1.01 (0.01)	0.008
Affymetrix	1	158.9	tsc0611403	1.93 (0.001)	0.88 (0.02)	**3.10 (0.00007)**	0.002
	2	4.1	tsc0943254	1.02 (0.01)	**2.75 (0.0002)**	0.19 (0.8)	0.005
	4	98.5	tsc0515689	0.95 (0.02)	0.23 (0.1)	**2.52 (0.0003)**	0.004
	7	28.0	tsc0309170	2.23 (0.0007)	0.14 (0.2)	**2.96 (0.0001)**	0.04
	11	128.2	tsc1017688	1.79 (0.002)	0.01 (0.6)	**3.31 (0.00004)**	0.008

^a^The markers which have a LOD score ≥ 2.5 and a parental effect *p *≤ 0.05 are listed. The highest LOD score at each location is in bold.

**Table 2:** Linkage results of multipoint analysis using sex-averaged genetic maps^a^

				LOD score (*p*-value)			
					
Marker data	Chr	Location (cM)	Closest marker	Both	Maternal	Paternal	Parental effect *p*-value
Microsatellite	2	78.0	D2S123	0.05 (0.3)	0.82 (1)	**1.71 (0.002)**	0.0007
Illumina	10	148.7	D10S1213	0.43 (0.08)	0.16 (0.8)	**1.67 (0.003)**	0.01
	12	161.6	rs1465727	0.74 (0.03)	**2.41 (0.0004)**	0.10 (0.7)	0.004
Affymetrix	13	89.8	rs1556800	0.38 (0.09)	0.07 (0.7)	**1.65 (0.003)**	0.01
	2	88.2	tsc0053926	0.62 (0.04)	0.002 (0.5)	**1.52 (0.004)**	0.04
	7	13.7	tsc1462560	1.34 (0.006)	1.01 (0.01)	**1.85 (0.002)**	0.008
	12	154.4	tsc0553175	0.71 (0.03)	**2.70 (0.0002)**	0.09 (0.7)	0.002

^a^The markers which have a LOD score ≥ 1.5 and a parental effect *p *≤ 0.05 are listed. The highest LOD score at each location is in bold.

**Table 3:** Linkage results of multipoint analysis using sex-specific genetic maps^a^

				LOD score (*p*-value)			
					
Marker data	Chr	Location (cM)	Closet marker	Both	Maternal	Paternal	Parental effect *p*-value
Microsatellite	12	153.7	D12S2078	0.49 (0.07)	**1.56 (0.003)**	0.10 (0.7)	0.02
Illumina	7	1.2	rs1362136	0.87 (0.02)	0.54 (0.06)	**1.52 (0.004)**	0.02
	9	59.0	rs954779	0.07 (0.3)	**1.66 (0.003)**	0.30 (0.9)	0.003
Affymetrix	10	57.0	rs927099	0.64 (0.04)	**1.55 (0.004)**	0.04 (0.3)	0.04
	12	161.6	rs1465727	0.91 (0.02)	**1.79 (0.002)**	0.09 (0.7)	0.03
	13	93.1	rs1415055	0.62 (0.04)	0.0002 (0.5)	**1.61 (0.003)**	0.03
	7	13.7	tsc1462560	1.26 (0.008)	0.85 (0.02)	**1.76 (0.002)**	0.01
	10	62.5	tsc0608195	0.92 (0.02)	**1.87 (0.002)**	0.009 (0.4)	0.04
	12	154.4	tsc0553175	0.71 (0.03)	**2.16 (0.0007)**	0.04 (0.6)	0.009

^a^The markers which have a LOD score ≥ 1.5 and a parental effect *p *≤ 0.05 are listed. The highest LOD score at each location is in bold.

## References

[B1] Song J, Koller DL, Foroud T, Carr K, Zhao J, Rice J, Nurnberger JI, Begleiter H, Porjesz B, Smith TL, Schuckit MA, Edenberg HJ (2003). Association of GABA(A) receptors and alcohol dependence and the effects of genetic imprinting. Am J Med Genet B Neuropsychiatr Genet.

[B2] Paterson AD, Petronis A (1999). Sex-based linkage analysis of alcoholism. Genet Epidemiol.

[B3] Strauch K, Fimmers R, Windemuth C, Hahn A, Wienker TF, Baur MP (1999). Linkage analysis with adequate modeling of a parent-of-origin effect. Genet Epidemiol.

[B4] Wyszynski DF, Panhuysen CIM (1999). Parental sex effect in families with alcoholism. Genet Epidemiol.

[B5] Page GP, King TM, Barnholtz JS, de Andrade M, Peterson LE, Amos CI (1999). Genome scans for genetic predisposition to alcoholism by use of transmission disequilibrium test analyses. Genet Epidemiol.

[B6] Kong X, Murphy K, Raj T, He C, White PS, Matise TC (2004). A combined linkage-physical map of the human genome. Am J Hum Genet.

[B7] Kong A, Gudbjartsson DF, Sainz J, Jonsdottir GM, Gudjonsson SA, Richardsson B, Sigurdardottir S, Barnard J, Hallbeck B, Masson G, Shlien A, Palsson ST, Frigge ML, Thorgeirsson TE, Gulcher JR, Stefansson K (2002). A high-resolution recombination map of the human genome. Nat Genet.

[B8] Gudbjartsson DF, Jonasson K, Frigge ML, Kong A (2000). Allegro, a new computer program for multipoint linkage analysis. Nat Genet.

[B9] Kong A, Cox NJ (1997). Allelic-sharing models: LOD scores and accurate linkage tests. Am J Hum Genet.

[B10] Karason A, Gudjonsson JE, Upmanyu R, Antonsdottir AA, Hauksson VB, Runasdottir EH, Jonsson HH, Gudbjartsson DF, Frigge ML, Kong A, Stefansson K, Valdimarsson H, Gulcher JR (2003). A susceptibility gene for psoriatic arthritic maps to chromosome 16q: evidence for imprinting. Am J Hum Genet.

